# Resilience-culture of support

**DOI:** 10.1192/bjo.2021.396

**Published:** 2021-06-18

**Authors:** Nazish Hashmi, Sunitha Muniyappl

**Affiliations:** 1 Leeds and York NHS Partnership foundation trust; 2 Bradford NHS foundation trust

## Abstract

**Aims:**

Better-informed trainees will have increased confidence and improved resilience which will have a positive impact on the workforce. To promote and celebrate diversity in psychiatric workforce it is imperative to acknowledge the above and provide adequate support to IMGs across UK.

**Background:**

Nearly two fifth of licensed doctors in NHS are from black and ethnic minorities. Studies have shown that International Medical Graduates (IMGs) are particularly prone to certain difficulties compared to UK graduates. IMGs are more likely to be subject to investigations by General Medical Council for concerns over clinical skills and knowledge, communication skills, lack of awareness of the laws and code of practice. This has been highlighted by GMC as well as Royal College of Psychiatrists. To promote and celebrate diversity in psychiatric workforce it is imperative to acknowledge this and provide adequate support to IMGs across UK.

**Method:**

An additional rotation wide induction programme was started for IMGs in August 2018 in West Yorkshire. This has continued on a 6 monthly basis for all new starters and last one was held on 21st of August 2019. Teaching included information about Good Medical Practice, confidentiality issues, principles of consent, information about living skills and practical teaching on phlebotomy and requesting investigations.

**Result:**

The doctors who attended these sessions found it to be very helpful and some suggested it to be a full day programme. According to the feedback collected there was a definite improvement in understanding noted by IMGs in most areas covered. This induction was also acknowledged in the School of Psychiatry conference in October 2019.

**Conclusion:**

Considering the increasing numbers of International medical graduates it will be beneficial to arrange similar events at local level for easier accessibility. In line with RCPsych and GMC guidelines all trusts should be encouraged to offer IMG induction sessions locally.

